# Defining feasibility and pilot studies in preparation for randomised controlled trials: using consensus methods and validation to develop a conceptual framework

**DOI:** 10.1186/1745-6215-16-S2-O87

**Published:** 2015-11-16

**Authors:** Sandra Eldridge, Christine Bond, Mike Campbell, Sally Hopewell, Lehana Thabane, Gill Lancaster, Claire Coleman

**Affiliations:** 1 Queen Mary University of London, London, UK; 2 University of Aberdeen, Aberdeen, UK; 3 Sheffield University, Sheffield, UK; 4 Oxford University, Oxford, UK; 5 McMaster University, Hamilton, Canada; 6 Lancaster University, Lancaster, UK

## 

Pilot and feasibility studies underpin much of current health related research, including randomised controlled trials. The number of reports in which authors describe their studies as pilot or feasibility studies is increasing. In spite of recent attempts to define pilot and feasibility studies, the research community has differing views about these definitions.

We will present a framework for defining pilot and feasibility studies conducted in advance of randomised controlled trials designed to assess the effect of an intervention. The framework was developed using: a Delphi consensus study (93 participants); an open meeting at the 2nd International Clinical Trials Methodology Conference in 2013 (15 participants); a review of definitions outside the health research context; an international expert consensus meeting (26 participants); and a review of published empirical studies described as pilot or feasibility (27 studies).

The framework uses feasibility as an umbrella term with pilot studies as a subset of feasibility studies; it does not use mutually exclusive definitions of pilot and feasibility studies. The framework involves straightforward definitions and can be represented in a simple diagram. We will show how it is consistent with the common usage of these terms in the literature, and with the UK Medical Research Council framework for complex interventions. Additionally although the UK National Institute for Health Research does imply that feasibility and pilot studies are mutually exclusive, their definitions can be mapped onto the framework. This work is part of a larger programme of work on pilot and feasibility studies.

